# Supplementary material for: Osteoporosis: A Small-Group Case-Based Learning Activity
Source: MedEdPORTAL. 2021 Aug 30;17:11176. doi: 10.15766/mep_2374-8265.11176 (PMC8403690; doi:10.15766/mep_2374-8265.11176)
Supplement: Supplementary file 1 — CBL Facilitator Guide.docxFace-to-Face Session Student Guide.docxRemote Learning Session Guide.pptxExam Question Descriptions.docxPostsession Survey.docx [file mep_2374-8265.11176-s001.zip › A. CBL Facilitator Guide.docx]

**FACILITATOR GUIDE**

**Case Content Cover Sheet**

| **Organ System** | MSK |
| --- | --- |
| **Case** | Osteoporosis |
| **Clinical Skills/Reasoning** |  |
|  | Obtain a history |
|  | Oral Presentation |
|  | Choose Focused Exam(s) |
|  | Examine each other (MSK) |
|  | Interpret Physical Exam |
|  | Choose diagnostic studies |
|  | Generate treatment plan |
|  | Generate a Problem List |
|  | Write a Prescription |
| **Interpretation of Studies** |  |
|  | Wrist X-ray |
|  | DXA |
| **EBM** |  |
|  | Absolute risk difference and number need to harm |
|  | Clinical Prediction Rule (FRAX) |
| **Community-Engaged** |  |
|  | Elderly Rx Safety |
| **Pharmacology** |  |
|  | Bisphosphonate, Calcitonin |
|  | Denosumab, Teriparitide |
|  | Raloxifene |

**FACILITATOR GUIDE**

**EDUCATIONAL OBJECTIVES**

1. Perform or observe an oral case presentation and complete a musculoskeletal physical exam after obtaining hypothesis driven medical history.
2. Given a history of an acute injury to the wrist, use exam and x-ray appearance to diagnose a Colles type distal radial fracture.
3. Define a fragility fracture and recognize osteoporosis as the most common underlying cause.
4. When provided with a DXA result and T-Score, interpret the results of a DXA scan and understand the meaning of T-Score ranges.
5. Use the FRAX tool to predict the ten-year risk of osteoporotic fracture in a patient.
6. Name the available pharmacologic treatments for osteoporosis and describe the mechanism of action and major adverse effects of each.

**EXECUTIVE SUMMARY**

This is a case of a 69-year-old woman who presents to the Emergency Room with a distal radial fracture sustained by a mechanical fall. The patient has several risk factors for osteoporosis but is not being treated for osteoporosis because she was found to have osteopenia by DXA scan several years earlier. Students will discover that her ten-year risk of fracture by FRAX Score was high enough to warrant treatment. They will work through the diagnosis of the fracture and move on to discuss her risk factors for osteoporosis, fracture prevention methods, and pharmacologic treatments.

Yellow highlighted text indicates facilitator notes as well as main focus areas for facilitator’s to focus probing questions.

**PART 1**

**Ms. Schick is a 69-year-old woman presenting to the Emergency Room with right wrist pain after a fall.**

**Q1. Is there any additional information that you want to ask of Ms. Schick? In addition to the group scribe, please take notes individually. After the history is obtained, one student will be asked to orally present the information that has been gathered.**

***Facilitators:*** *Below are some questions and “answers” that the students may wish to ask. If they ask a question not listed below, use your best judgment in answering. You can always reply “that information is not available at this time” or “I don’t know.” Provide answers only to the questions asked. You may need to push them along to think more broadly about questions – they should at least hit all of the highlighted questions.*

*If they haven’t already assigned someone, encourage the students to choose a group scribe to record important information on the board.*

| **QUESTIONS TO ASK** | **Answers** |
| --- | --- |
| Circumstances of the fall, what happened? | It’s so silly, really. I was leaving Publix this afternoon, and on my way back from returning the cart to the front, I guess I tripped over one of those concrete parking stop things…I didn’t pass out or anything… |
| How did you land, did you hurt anything else? | I fell forward. I tried to break the fall with my arms, but that apparently didn’t work out so well. I fell on my right side, so my hip is a bit sore, but I didn’t hit my head or anything. Only if they ask about weight bearing or more about the hip: Oh yeah, I can walk. |
| When did the pain start? Other descriptors of pain (severity, quality, location, radiation, etc.) | I knew it was bad right away. The pain was just awful, 10/10 at the time, but it’s a little better now – maybe 5/10 if I don’t move it or touch it. It’s worst right here (point to distal end of the radius on your right arm, just proximal to the wrist), but I feel it all the way from my fingers to my elbow. |
| Other complaints about the arm (tingling, numbness, bleeding, range of motion) | It’s really just the pain. I guess I have some scrapes, but nothing too bad. No tingling, just lots of pain. I can’t really move my wrist or my fingers, it just hurts way too much to even try. |
| Syncope, loss of consciousness, seizure | No, I never fainted. I remember it all. I felt fine before – not dizzy or anything. |
| Past Medical History | If asked “general PMH”: I have high blood pressure. Otherwise, no. |
| Osteoporosis, osteopenia | I don’t think so…they did a test a few years ago and said I was ok… |
| Past Surgical History | I had a C-section when my son was born. He was upside down. |
| Allergies | None that I know of. |
| Medications | Just the blood pressure pill. Hydro-something? |
| Steroids | Not that I can remember. |
| OTC meds, vitamin, herbals | I’ve never been good at remembering, but I do try to take a multivitamin. Only if they ask about Calcium or vitamin D: If it’s in the multivitamin, I guess so. I don’t take any extra stuff. |
| Social History |  |

| Tobacco | Yeah…I know I need to quit. I’ve been trying for years. Only if they ask how much: About half a pack a day since I was in my twenties. I quit a bunch of times, but it always seems to catch up with me again. |
| --- | --- |
| EtOH | I have a glass of wine maybe once a week with dinner. |
| Drugs | Oh, no. Never. |
| Occupation | I used to be a teacher. Sixth grade math. But I retired last year. |
| Living situation | I live with my husband. My son lives nearby with his family – he’s got two girls. |
| Diet/Exercise | I’ve never really had a problem with weight. I guess I’m blessed like that. We eat ok, but we do a lot of take-out, so it’s not so healthy. My husband just joined a gym, but I really don’t like that kind of thing. I’m pretty active anyhow – running around town and with my grandbabies and all. |
| Family History | My mother died when she was in her 70s, I think she had a stroke. My father died a few years later, in his 70s, too. He had pneumonia, I think. |
| Family history of hip (or other) fracture | Only if they ask specifically about it: Yeah, my mom broke a hip not long before she died. They fixed it, but she ended up with so many complications. Eventually she had the stroke in rehab and that did her in. I think that hip fracture was kind of like the beginning of the end for her, though… |
| Sexual History | Oh, just my husband. We’ve been married for over 40 years. Only if f they ask about menopause: I think I was 46 or so? They put me on the hormones for a while, but my breasts were so sore that I had to stop. |

***Facilitators:*** *When they have completed asking questions, (should at least hit all of the highlighted ones on the left), please ask for a volunteer to orally present the history. They can use their notes or the notes on the board. The results will vary and there is no “correct” presentation. The written history that follows is a fair example.*

*The most important points that they should understand from the exercise is that the HPI “tells the story” in a complete and fairly chronological way and addresses all of the pertinent positives and pertinent negatives. They should also understand that a complete oral presentation follows the same format as the written history and includes all categories such as PMH, PSH, Meds, Allergies, etc. This is one of the first times we are asking them to do this, so if no one volunteers, pick someone who seem confident and most capable. This should be a positive, formative experience – do not expect them to be able to do this easily or well.*

*After briefly reviewing the presentation, you can give them part 2.*

**PART 2**

**HPI**

Sophie Schick is a 69-year-old woman with a history of hypertension who presents with right wrist pain after a mechanical fall. Earlier today, she tripped over a parking stop in a grocery store parking lot, falling on her outstretched right arm. She landed on her right side, striking her hip, but did not hit her head. She has some pain at the hip, but has no trouble walking or weight bearing. She felt fine before the fall, denies dizziness or lightheadedness, and did not lose consciousness before or during the event. She felt immediate severe 10/10 pain in her right wrist which has now subsided to a 5/10. The pain is localized to the distal end of the right forearm, but radiates down to the fingers and up to the elbow. She is unable to move her wrist or fingers due to the pain, but denies paresthesia.

**PMH**

She has a history of Hypertension.

She has no known history of osteoporosis or osteopenia – she was told by her doctor several years ago that a bone test was “normal.”

**PSH**

She had a C-section for the birth of her son.

**MEDS**

She takes a medication for hypertension. She does not recall the full name, but it sounds like it is probably hydrochlorothiazide. She also takes a multivitamin sporadically but no supplemental calcium or Vitamin D.

**ALLERGIES**

No known drug allergies

**SH**

She is married and lives with her husband. Her son and his family live nearby. She worked as a 6^th^ grade math teacher but retired last year. She smokes about ½ a pack of cigarettes daily and has done so on and off since her 20s, but is interested in quitting. She drinks about 1 glass of wine per week with dinner and has no history of drug use. Her diet consists primarily of prepared foods and while she is active, she does not do any form of regular exercise.

**FH**

Her mother died in her 70s from a stroke that occurred during a prolonged and complicated recovery from a hip fracture. Her father also died in his 70s from pneumonia.

***Facilitators:*** *Take a moment for the students to reflect on anything in the written history that they didn’t ask about on their own. Also remind them how the written history is a decent template for the oral presentation.*

**Q2. Which parts of the focused physical exam would you perform? Why?**

Vitals: Vitals are always vital!

BMI: low body weight is a risk factor for osteoporosis

General: Always important to assess, get first impressions

HEENT: Look for signs of head trauma.

MSK: Exam of right arm. Also complete MSK exam to look for other areas of trauma. Special points to consider:

- Palpation of spine for point tenderness (acute fracture) as well as ROM and palpation that might reveal limitation or loss of height (old vertebral fracture)
- ROM and palpation of the hips (especially right side where she fell).
- It is always important to inspect the joints above and below the injury, too.
- Checking for tenderness in the anatomic snuffbox is important because fracture of the scaphoid is also often caused by a fall on an outstretched hand – this fracture is often not seen on x-ray.

Cardiac: May be important for possibility of syncope as a cause for the fall. Definitely want to check distal pulses in the right arm.

Pulmonary: May be important if you are concerned about undiagnosed COPD because of the smoking history.

Neuro: Assess for sensation of right hand/arm. Also may be important for possibility of syncope or seizure as a cause for the fall.

Skin: Evaluate for abrasions, ecchymoses, or other signs of trauma.

***Facilitators:*** *Don’t need to belabor this point. They should come up with at least the highlighted areas. Most importantly, push them for what it is they are looking for on exam. After they have discussed completely (but before you give them part 3, ask for a volunteer to perform the complete MSK exam (without the checklist!) on another student. Have the other students follow along on the attached checklist. Once the first student has done as much as they can remember, let the group correct any errors and ask for another student to complete the exam.*

| **MUSCULOSKELETAL EXAM** | **NOT DONE** | **DONE INCORRECTLY** | **DONE CORRECTLY** | **COMMENTS** |
| --- | --- | --- | --- | --- |
| **Professionalism** | | | | |
| 1. Washes hands |  |  |  |  |
| 1. Introduces self and explains role |  |  |  |  |
| 1. Explains to patient what he/she is going to do |  |  |  |  |
| 1. Attentive to comfort throughout the exam - *(i.e. asks permission to expose or touch, assists pt on and off the exam table).* |  |  |  |  |
| **Head** | | | | |
| 1. Assesses TMJ - *place tips of fingers anterior to the tragus of each ear, asks pt to open mouth.* |  |  |  |  |
| **Shoulder** | | | | |
| 1. Inspects - *fully exposes the shoulder and verbalizes inspection or states what they are looking for on exam (i.e. “I’m looking for any swelling, deformities, skin lesions, etc.)* |  |  |  |  |
| 1. Palpates - *demonstrates thoughtful palpation of the following areas: sternoclavicular joint, clavicle, AC joint, scapula, coracoid process, greater tubercle of the humerus, biceps groove, subacromial area, and muscles.* |  |  |  |  |
| 1. Assesses ROM - *instructs patient to perform the following movements: flexion, extension, external and internal rotation, abduction and adduction.* |  |  |  |  |
| **Elbow** | | | | |
| 1. Inspects - *verbally indicates inspection of elbows.* |  |  |  |  |
| 1. Palpates - *demonstrates thoughtful palpation of the following areas: ulna, olecranon, ulnar groove, lateral and medial epicondyles.* |  |  |  |  |
| 1. Assesses ROM - *flexion, extension, supination and pronation.* |  |  |  |  |
| **Wrist and Hands** | | | | |
| 1. Inspects - *verbally indicates inspection of wrists, hands, & fingers.* |  |  |  |  |
| 1. Palpates - *both wrists, MCP, PIP, and DIP joints*   *Student should palpate each MCP joint on the sides of the knuckles. DIP and PIP joints should be palpated using 4 fingers (thumb and index finger over medial/lateral aspects, thumb and index finger over superior and posterior aspects).* |  |  |  |  |
| 1. Assesses ROM   *wrists - flexion/extension, lateral and medial movement*  *fingers - flexion/extension and adduction/abduction* |  |  |  |  |
| **Hips** | | | | |
| 1. Inspects and Palpates - *verbally indicates that he/she would inspect and palpate the hips (they are not expected to expose or touch the patients’ hips during the OSCE).* |  |  |  |  |
| 1. Assesses ROM - *Flexion, internal/external rotation, abduction/ adduction, and extension (these movements can be performed with the patient lying, sitting, or standing. Student should provide clear instructions and assist with position changes, tries to limit the number of times the pt must get on and off the exam table).* |  |  |  |  |
| **Knees** | | | | |
| 1. Inspects - *verbally indicates inspection of the knees.* |  |  |  |  |
| 1. Palpates - *performs thoughtful palpation of the patella, anterior, medial, lateral, and posterior aspects of the knee.* |  |  |  |  |
| 1. Assesses ROM - *flexion and extension* |  |  |  |  |
| **Ankles and Feet** | | | | |
| 1. Inspects - *verbally indicates inspection of the ankles and feet.* |  |  |  |  |
| 1. Palpates - *student should palpate both ankles and MTP joints.* |  |  |  |  |
| 1. Assesses ROM - *dorsiflexion, plantarflexion, inversion, eversion.* |  |  |  |  |
| **Spine** | | | | |
| 1. Inspects - *student asks permission to fully expose the patient’s entire back and verbally indicates inspection of the spine*. |  |  |  |  |
| 1. Palpates - *student should palpate the spinous processes from the cervical spine all the way down to the lumbar spine. Then palpate the paravertebral muscles in several areas bilaterally.* |  |  |  |  |
| 1. Assesses ROM of the neck - *flexion, extension, rotation, and lateral bending.* |  |  |  |  |
| 1. Assesses ROM of the back - *flexion, extension, rotation, and lateral bending.* |  |  |  |  |

**PART 3**

**Physical Examination**

Vitals:

Temp: 37.6 C HR: 97 bpm RR: 16 bpm BP: 142/84 SaO_2_ 98% on Room Air Weight: 53 kg Height: 5’3”

General: Pleasant, thin woman sitting up on the edge of the stretcher in mild-moderate pain.

HEENT: Atraumatic. Pupils equal, round, and reactive to light and accommodation. Sclerae clear, conjunctivae pink. Mucus membranes moist.

Chest: Clear to auscultation bilaterally.

CV: Normal S1S2, no murmurs/rubs/gallops.

Skin: Warm and dry. Scattered excoriations over the palms bilaterally and ecchymoses seen over the right hip.

Neuro: Alert and oriented, CN II-XII intact, normal strength and tone in all extremities except RUE, normal gait.

MSK: Thin with low-normal muscle bulk. Except for RUE, all joints have full range of motion without swelling, tenderness or erythema. There is no point tenderness to palpation along the length of the spine and no obvious kyphosis or loss of height. The RUE is normal above the wrist, including the elbow. Joints of the hand (MCPs, DIPs, and PIPs) are normal. There is no tenderness to palpation of the “anatomic snuffbox.” Just proximal to the wrist, the arm is warm, edematous, and grossly deformed as shown below:


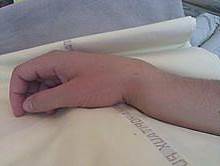


Image by Sylvian Letuffe, retrieved from: <https://commons.wikimedia.org/wiki/File:Poignet_Gauche_suite_a_fracture_type_Pouteau_Colles.jpg> on 12/11/2018. Image is in the public domain.

**Q3. How do you interpret the results of the physical exam?**

- The vital signs are normal. The students should calculate her BMI (21) and realize that it is low normal.
- She is slight in appearance and Caucasian, both risk factors for osteoporosis.
- Mucus membranes are moist suggesting that orthostasis due to volume depletion is unlikely.
- The cardiac, pulmonary, and GI exams are unremarkable, making other systemic illness somewhat less of a concern.
- The normal pulses and no neurologic deficits are reassuring that she didn’t sustain any neurovascular injury.
- Ecchymoses on the dermatologic exam help pinpoint areas like the hip that may have also sustained injury and helps to rule out any open fractures.
- The picture of the hand suggests distal radial fracture with dorsal displacement of the hand (which will be discussed next).

**Q4. What is the significance of the fact that there was no tenderness to palpation of the “anatomic snuffbox?” What are the borders of the “anatomic snuffbox?”**

The “anatomic snuffbox” is a depression bordered by the tendons of the extensor policis longus, extensor policis brevis, and abductor policis longus.

Pain on palpation of that area is a highly sensitive test for fractures of the scaphoid bone of the wrist. This type of fracture is also caused by falling on an outstretched hand and can be missed on x-ray.


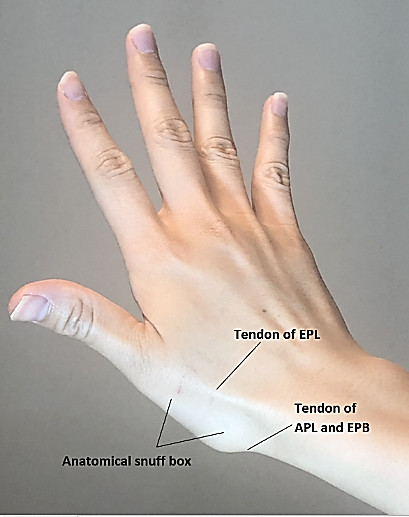

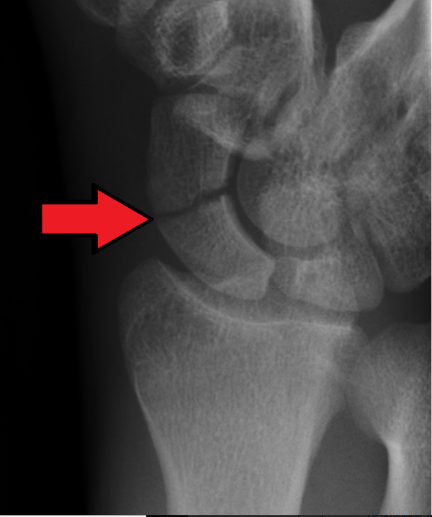

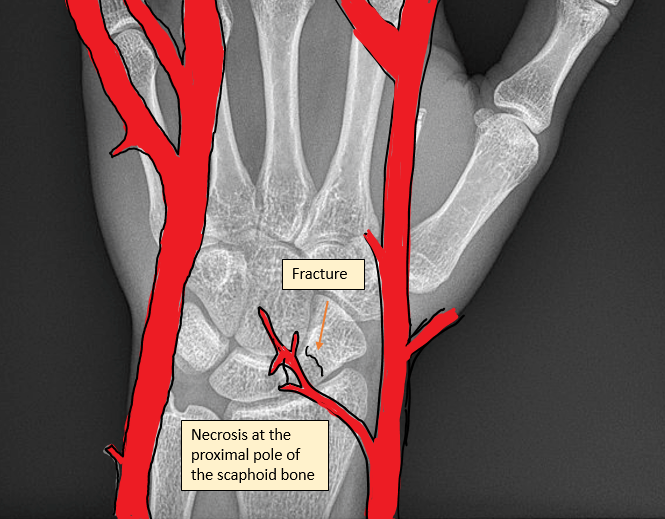
The blood supply to the scaphoid comes from the radial artery so the proximal portion has no direct blood supply. If the fracture isn’t recognized and repaired, it may lead to avascular necrosis.

1. Author Owned

2. Image by Gilo1969, retrieved from: <https://commons.wikimedia.org/wiki/File:CollesfractureArrow.png> on 12/11/2018. Creative Commons License associated: <https://creativecommons.org/licenses/by/3.0/deed.en>

3. Author Owned

**Q5. What type of injury did Ms. Schick most likely incur by falling on her extended wrist? Describe some of the clinical characteristics suggestive of the injury.**


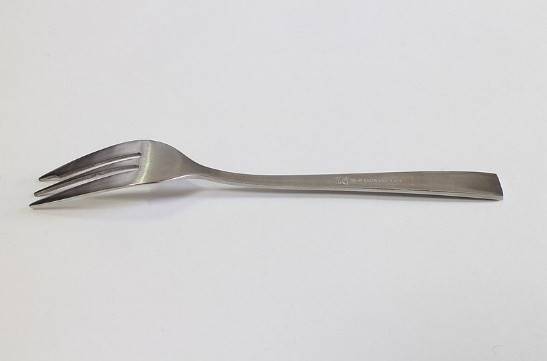


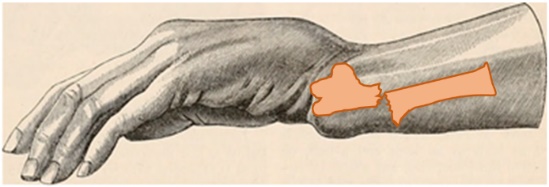
This is most likely a Colles Fracture. The term “Colles Facture” technically means a fracture of the distal radius with the classic deformity seen above. That said, it is often used to describe any fracture of the distal radius with the deformity shown, with or without concomitant fracture of the distal ulna.

The fracture can usually be recognized clinically by the classic deformity – often described as “dinner fork” or “bayonet” deformity because the hand is displaced dorsally and tilts radially due to dorsal displacement of the fracture fragment (as shown here).

1. Image by Toddy, Robert Bentle, retrieved from: [hthttps://commons.wikimedia.org/wiki/File:%22Dinner_fork%22_Deformity.jpg](https://commons.wikimedia.org/w/index.php?search=dinner+fork+deformity&title=Special:Search&go=Go#/media/File:%22Dinner_fork%22_Deformity.jpg) on 12/11/2018. Image is in the public domain.
2. Image by Yapparina, retrieved from: https://commons.wikimedia.org/wiki/File:18-8_stainless_steel_fork_2.jpg on 12/11/2018. In the public domain.

A Colles Fracture is most often caused by falling on an outstretched hand and is one of the most common fractures seen in patients with osteoporosis.

Ms. Schick’s case is highly suggestive of a Colles Fracture. She is a petite woman with many risk factors for osteoporosis (we will list them shortly), she fell on an outstretched hand, and she has the classic deformity.


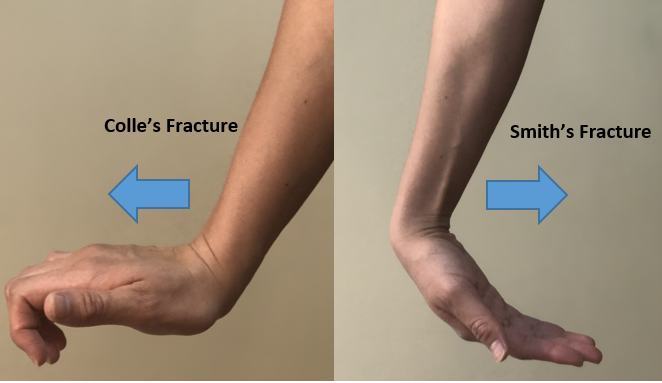
**Q6. What if Ms. Schick had flexed her wrist prior to falling, which other type of injury might you expect her to have sustained?**

The Colles fracture (dorsal displacement of the hand) is caused by falling on to an outstretched arm with the wrist extended to break the fall (the natural instinct). The opposite (ventral displacement of the hand) occurs when falling on a flexed wrist. This type of injury is called a Smith’s Fracture.

Author owned.

**Q7. Based on the history and physical exam, what other studies/interventions might you initiate in the ER today and why?**

***Facilitators:*** *Specific information and directions are included in each bullet below.*

Some things the students might suggest (nudge them until they get the highlighted items):

- Plain radiographs of the wrist: Ask them why. How many views? They don’t need to get this exactly, but they should understand that more than one view is necessary. If they just did anteroposterior views, they could not reliably say if there was any dislocation dorsally or ventrally (in the same plane). If they just did lateral, they could not clearly evaluate the long bones because they would be superimposed on one another.
- Plain radiographs of the right hip: Ask why. They SHOULD do this. She struck her hip in the fall and has ecchymoses on exam. She doesn’t have any trouble weight bearing, but is certainly at risk for hip fracture.
- CT of the head: Why? Not 100% indicated (she denies hitting her head and has no evidence of trauma), but certainly not unreasonable and almost certainly would be done in most modern ERs to rule out emergent injuries like subdural hematoma.
- Chem7: Why? Not 100% necessary, but reasonable. To look for electrolyte abnormalities that may have contributed to a fall or to an underlying risk of osteoporosis. Especially calcium levels.
- CBC: Why? Reasonable. To look for anemia, which might indicate malabsorption or other process.
- Serum Vitamin D level: Again, not unreasonable, but would not be a test done by physicians in the emergency room.
- CXR: Ask them why, what are they looking for? Not really needed. Maybe reasonable if they were looking for rib fractures or hyperexpansion due to COPD.
- EKG: Ask them why. Might be reasonable if they’re considering cardiogenic syncope due to arrhythmia.

**PART 4**

**Plain radiographs of the right wrist are performed (as seen). Radiographs of the right hip were also performed and showed no evidence of fracture. A CT of the head was performed and showed no evidence of trauma or intracranial hemorrhage.**


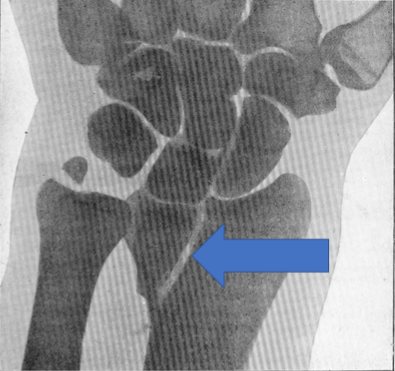


Image by Wikimedia Commons retrieved from: <https://commons.wikimedia.org/w/index.php?curid=36355446> on 11/03/2020. Creative Commons License associated: <https://creativecommons.org/licenses/by/4.0/deed.en>

**Q8. How do you interpret the x-ray? What type of fracture did Ms. Schick sustain? Use descriptive words to accurately describe the injury (i.e. open vs. closed, intra- vs. extra-articular, displaced vs. non-displaced, transverse vs. oblique vs. comminuted, etc.).**

This is a Colles Fracture of the distal radius.

- The fracture is **closed** because the skin overlying the fracture is intact.
- It is **extra-articular** because the fracture does not run through the articular surface of the bone involving the articular cartilage (see figure below).
- It is **displaced** because the fracture fragment is no longer in proper anatomic alignment with the remainder of the bone.
-
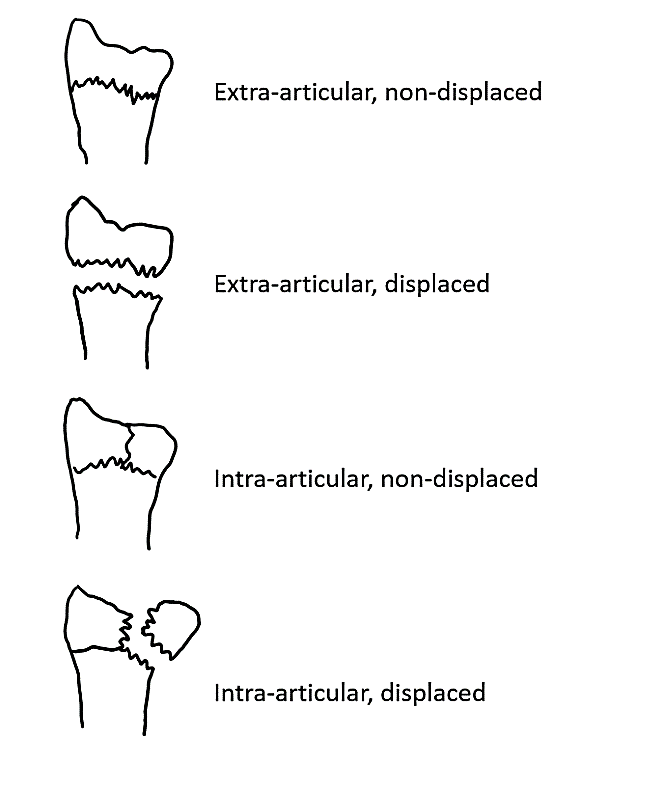

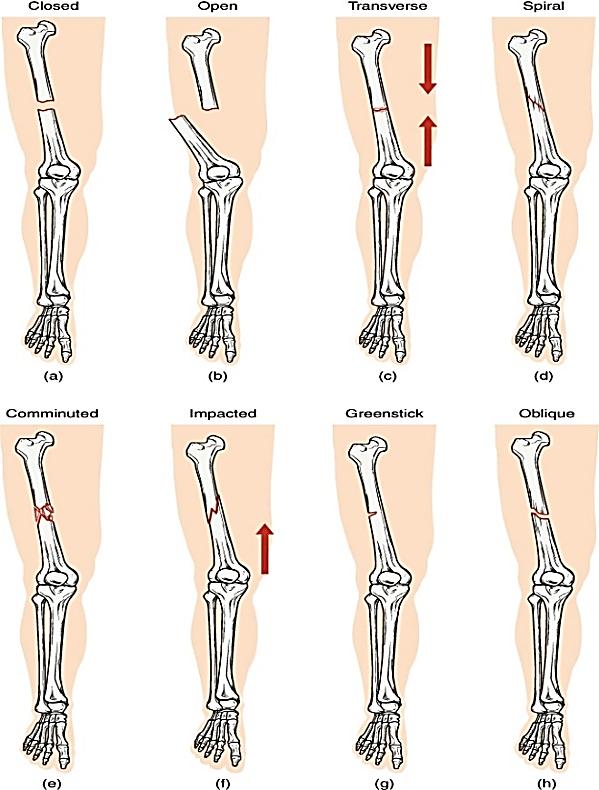
The fracture is **oblique** because the fracture line is oblique to the axis of the bone. Students may say transverse – which is not entirely incorrect, but transverse implies that the fracture line is perpendicular to the long axis of the bone. The figure below shows some of the common fracture patterns. If students suggest other patterns, probe them further – ask them to draw several of the patterns shown below.

1. Author owned.

2. Image by OpenStax College, retrieved from: <https://commons.wikimedia.org/wiki/File:612_Types_of_Fractures.jpg> on 01/08/2019. Creative Commons License associated: <https://creativecommons.org/licenses/by/4.0/deed.en>

**Q9. Based on the clinical presentation, are you concerned that Ms. Schick may have suffered a pathologic fracture? If so, which type is most likely? Define the term and explain your answer. In order to further investigate, which test(s) would you recommend that her primary care physician perform?**

A pathologic fracture is one that occurs in an area of bone that is weakened by another disease process. Fragility fractures, a subtype and the most common form of pathologic fractures, most often occur in the setting of osteoporosis.

Ms. Schick has many risk factors for osteoporosis (we are about to list them) and the distal radius is one of the most common sites for osteoporosis related pathologic fractures. Bone densitometry by DXA should be performed.

**PART 5**

**Ms. Schick tells you that she is pretty sure her doctor did similar tests three years ago. Her husband brings the following results from home (shown below).**


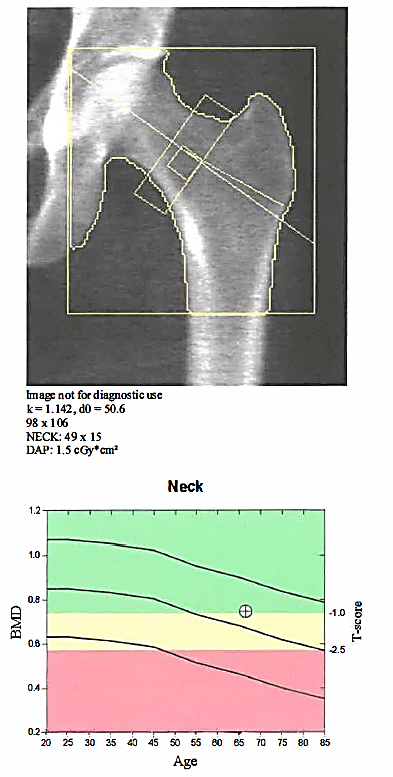
**
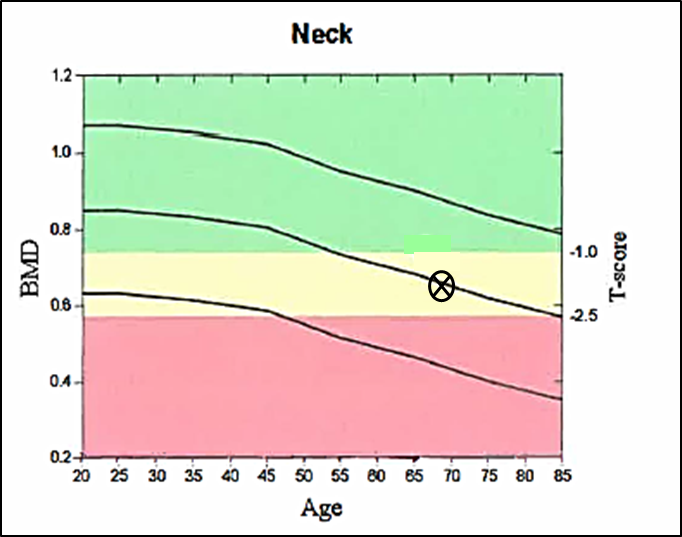
**

1. Author owned.

2. Author owned.

**Q10. What type of test is this? How do you interpret the results? What was Ms. Schick’s T-score? What does that mean?**

These are the results of a DXA (Dual Energy X-ray Absorptiometry) scan. The DXA scan measures bone mineral density (BMD) at several sites in the body (typically at the hip, lumbar spine, forearm, etc.). A patient’s BMD at a particular site (the hip, for example) is compared with the mean BMD of a gender matched and ethnicity matched control population. The Z-score (students do not need to discuss this – for your additional info in case they talk about it) is a comparison of the patient’s results to *age-matched* controls of the same gender and ethnicity. The T-score is a comparison of the patient’s results to the results of healthy 30-year-old controls of the same gender and ethnicity. *A T-score of > -1 (green) is considered normal. T-scores between -1 and -2.5 (yellow) represent osteopenia. T-scores < -2.5 (red) represent osteoporosis.*

The most important points to discuss here:

- This is a DXA scan, it measures BMD.
- What is a T-Score? A comparison of the patient’s results to the results of healthy 30-year-old controls of the same gender and ethnicity. (> -1 is normal, between -1 and -2.5 is osteopenia, < -2.5 is osteoporosis).
- What’s her T-score? Approximately -2.
- What does that mean? Consistent with osteopenia.

**Q11. Does Ms. Schick have osteoporosis? Did she have osteoporosis three years ago? Should she have received treatment three years ago? Why or why not?**

As discussed above, she had osteopenia three years ago by DXA. She has now sustained a fragility fracture (a fracture sustained by falling from standing height). Most experts would agree that she qualifies for treatment now. As for three years ago, this situation is a nice illustration of why **DXA alone should not be used to predict fracture risk in patients with significant clinical risk factors.**  To address this problem, USPSTF recommends using clinical risk assessment tools. There are few different tools available to assess osteoporosis risk. The FRAX tool (University of Sheffield) is a commonly used clinical assessment tool, which uses risk factors alone or risk factors with the results of DXA scanning to determine an individual’s 10-year risk of fracture.

This tool is available on-line (<http://www.shef.ac.uk/FRAX>, there are also office versions) and students should look it up now to calculate her risk. It is an option especially for clinically higher risk patients who may not have insurance coverage or otherwise be able to afford a BMD – to determine whether treatment should be initiated. (FRAX predictions have been validated on US populations even WITHOUT BMD data.)

Based on her individual risk factors, Ms. Schick had a predicted 10-year risk of 3.4% for hip fracture and 18% for other major osteoporotic fracture (like a distal radius fracture) three years ago. By current guidelines (which are also visible on the website, just below the calculation tool), she did qualify for treatment three years ago.

Furthermore, three years ago by clinical data alone (without the BMD) Ms. Schick had a predicted 10-year risk of 4.9% for hip fracture and 20% for other major osteoporotic fracture.

Current guidelines recommend treatment for patients who have sustained a fracture of the hip or vertebra (regardless of BMD), patients with osteoporosis by T-score < -2.5, and patients with a predicted 10-year risk of hip or other major osteoporotic fracture greater than 3% or 20%, respectively.

**PART 6**

**Q12. In retrospect, what risk factors did Ms. Schick have for an osteoporotic fracture? What are some other common risk factors?**

The most predictive risk factors are shown below. Ms. Schick’s risk factors are highlighted.

- Advanced age
- Previous fracture
- Long-term glucocorticoid therapy
- Low body weight (less than 58 kg [127 lb.])
- Family history of hip fracture
- Cigarette smoking
- Excess alcohol intake

Women are at greater risk than men.

In addition, certain ethnicities (Caucasians, Asians) are at increased risk when compared with other groups.

**Q13. In addition to the distal radius, Ms. Schick is at increased risk for fractures at which other sites?**

The most common fracture sites for patients with osteoporosis or osteopenia include:

- Hip
- Distal Radius
- Thoracic spine (compression)
- Proximal humerus

**Q14. How would you recommend that Ms. Schick be treated? If that includes pharmacologic interventions, be sure to describe the mechanism of action of the first line pharmacologic treatment and any important adverse effects and/or important patient instructions.**

***Facilitators:*** *Most importantly, they should discuss the bisphosphonates. Most important points are underlined.*

Bisphosphonates (such as alendronate, risedronate) are recommended for first line therapy because of their efficacy, favorable cost, and the availability of long-term safety data.

- - *Mechanism:* Bisphosphonates work by *slowing bone resorption*. They inhibit the action of osteoclasts and promote osteoclast apoptosis. In case students bring up a deeper micro level, they learned in pharmacology that: 1) non-nitrogen containing bisphosphonates compete with ATP in the osteoclast thus starving the cell and leading to apoptosis, and 2) nitrogen containing bisphosphonates inhibit the enzyme farnesyl pyrophosphatase synthase (FPPS) which is important for maintenance of the cytoskeleton of the osteoclast ruffled border leading to reduced bone resorption.
  - *Adverse effects:* One rare but major adverse effect is *osteonecrosis* of the jaw after dental surgery. Much more common is *reflux, esophagitis, and esophageal ulceration* which is why patients are instructed to take the medication with large volumes of water, i.e. at least 8 oz (to decrease the risk of tablet getting stuck in the esophagus) and sit upright for at least half an hour (to decrease the risk of reflux). Bisphosphonates are poorly absorbed orally. Because of that, patients are advised to take the medication first thing in the morning empty stomach, with a glass of water and wait at least 30 min before eating or taking any other medications to maximize the bisphosphonate absorption.

**Q15. What other (second- or third-line) pharmacologic treatment options might you consider? Be sure to describe the mechanism of action of each.**

- Raloxifene:
  - *Mechanism:* Raloxifene is a selective estrogen receptor modulator (*SERM*). Raloxifene has a partial agonist effect on bone where it exerts an *antiresorptive effect*, has antagonist effects in breast tissue where it reduces the incidence of breast cancer and has no estrogenic effects on endometrial tissue in postmenopausal women.
- Denosumab:
  - *Mechanis*m: *Inhibits bone resorption.* This is a *monoclonal antibody against RANKL* (RANK ligand). Osteoclasts express the RANK receptor on their surface. When the receptors bind free RANKL, it leads to proliferation and maturation of preosteoclasts into mature osteoclasts. This drug gobbles up free RANKL so that RANKL cannot bind to its receptor.
- Calcitonin:
  - *Mechanism*: Works to decrease serum calcium levels by *inhibiting osteoclastic bone resorption*.
- Teriparatide:
  - *Mechanism:* This is a *recombinant form of human parathyroid hormone* (*PTH)*. While it sounds counterintuitive, once daily subcutaneous administration of teriparatide results in *stimulation of osteoblastic activity resulting in new bone formation* in contrast to other drugs used in the treatment of osteoporosis which inhibit bone resorption.

**Q16. In addition to pharmacologic interventions, what could Ms. Schick have done to mitigate her risk of sustaining an osteoporotic fracture? What factors will you tell her to consider now? Consider lifestyle choices as well as other environmental factors.**

- Exercise: Especially weight bearing or resistance exercises. Exercises that improve balance (like yoga or Tai Chi).
- Diet: Recommendation is 1,200 mg/day of Calcium and 800 IU/day of Vitamin D. Foods like dairy products or leafy vegetables contain high levels of calcium. Usually oral supplementation is needed to meet recommended goals.
- Smoking: Stop.
- EtOH: Limit to <3 drinks daily.
- Prevent falls: Eliminate area rugs in the home (easy to trip on edges or slide), tie down loose electrical and other cords, adequate lighting in the house (especially by steps, entryways), use bars in the shower and bath, have vision checked regularly and update glasses if needed, wear comfortable flat shoes with good tread, reconsider medications that may cause sedation or orthostasis.

*Facilitators: Please make sure that the students talk about the things to ask about in the home, medications, etc. They will likely get the diet and exercise part, but we would like them to also start thinking about the additional risk factors that they should discuss with patients.*

**Q17. Please generate a problem list for Ms. Schick. Take a moment to do this individually, and then discuss together as a group.**

A sample problem list for this patient might look like this:

- Right wrist fracture (could be more specific like Colle’s or distal radial)
- Osteoporosis
- Hypertension
- Tobacco use
- Poor diet/exercise

Students may come up with other “valid” problems to include on the list, but it should at least have all of those listed above. If they are missing important problems, you can refer them back to the original history to read more closely and see if they pick up on the additional issues.

Students often miss problems seemingly unrelated to the HPI (chronic illnesses, social issues like tobacco use, financial issues, poor diet/exercise), and familial risk factors (like early CAD or cancer). Please remind them that anything they will need to address (anything that will require a plan, intervention, referral, counseling, etc.) should be on the problem list. In this case, an assessment of her willingness to discuss tobacco cessation and a discussion about diet/exercise would be warranted.

**PART 7**

**You discuss treatment options and your recommendation for alendronate treatment with Ms. Schick. She expresses concerns about potential adverse effects, stating “I saw a report on breakdown of the jaw that can occur from medications used to treat osteoporosis. Is this the same medication?” When you tell her that it is, she enquires about the risk of this complication. In order to answer her question, you find a recent systematic review that addresses the risk of osteonecrosis of the jaw with bisphosphonate treatment (data summarized below). The same data is presented below in a tabular format.**

| **Patient population** | **Incidence of Osteonecrosis of the Jaw** | | **ARD** | **NNH** |
| --- | --- | --- | --- | --- |
|  | **Receiving**  **Bisphosphonates** | **Not Receiving Bisphosphonates** |  |  |
| **Patients with *osteoporosis* receiving *oral* bisphosphonates** | 0.01% | 0.001% |  |  |
| **Patients with *cancer* receiving *intravenous* bisphosphonates** | 7.5% | 0.001% |  |  |

**Q18. Calculate the absolute risk difference (ARD) and the number needed to harm (NNH) for the two patient populations included in this study. How would you interpret and present this data to Ms. Schick?**

| **Patient population** | **Incidence of Osteonecrosis of the Jaw** | | **ARD** | **NNH** |
| --- | --- | --- | --- | --- |
|  | **Receiving Bisphosphonates** | **Not Receiving Bisphosphonates** |  |  |
| **Patients with *osteoporosis* receiving *oral* bisphosphonates** | 0.01%  (0.0001) | 0.001%  (0.00001) | 0.009%  (0.00009) | 11,111 |
| **Patients with *cancer* receiving *intravenous* bisphosphonates** | 7.5%  (0.075) | 0.001%  (0.00001) | 7.499%  (0.075) | 13.3 |

- The incidence of osteonecrosis of the jaw in patients with osteoporosis not receiving a bisphosphonate is 1/100,000 (0.00001 or 0.001%).
- The incidence of osteonecrosis of the jaw in patients with osteoporosis treated with oral bisphosphonates is ten-fold higher, i.e. 1/10,000 (0.0001 or 0.01%).
- The ARD is calculated as the difference between the incidence of treated and untreated patients. For patients with osteoporosis, this difference is 0.0001 – 0.00001 = 0.00009.
- The NNH is the reciprocal of the ARD (1/ARD), or 1/0.00009 = 11,111. This is a very high number needed to harm, indicating that you would need to treat more than 11,000 patients to cause one additional case.

In contrast, for patients with cancer treated with high-dose IV bisphosphonates, the incidence of osteonecrosis of the jaw is much higher (0.075 or 7.5%), and the NNH of 13.3 is much lower.

This data indicates that osteonecrosis of the jaw is primarily an adverse effect seen in cancer patients treated with IV bisphosphonates, with only a very small increase in risk for patients with osteoporosis treated with oral bisphosphonates.

In presenting the data to Ms. Schick, students should use natural frequencies (i.e., 1 in 1000, 1 in 10,000, etc.) and avoid use of fractions or percentages. They should clearly explain that the large NNH for patients with osteoporosis represents a very small (even trivial) increase in risk that is not clinically significant. This small risk would not outweigh the benefits of the medication in decreasing future fracture risk for Ms. Schick. That said, the patient should be an active participant in the conversation and ultimately the decision maker regarding her care.

(For additional information, please refer to this article which was used to extract the data. Khosla S, Burr D, Cauley J,et al. [Bisphosphonate-associated osteonecrosis of the jaw: report of a task force of the American Society for Bone and Mineral Research.](https://www.ncbi.nlm.nih.gov/pubmed/17663640/)J Bone Miner Res. 2007 Oct;22(10):1479-91. doi: 10.1359/jbmr.0707onj. PubMed PMID: 17663640.)

**Q19. You decide to treat Ms. Schick with alendronate. Her DOB is 1/31/49. Using the prescription pad below, write a prescription for this medication. You may use electronic devices to help you decide upon a dose, frequency, and route of administration.**

**For** Sophie Schick (DOB 1/31/49) **Date** Today

**Address** Patient Home Address


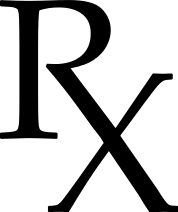
 Alendronate 70 mg

One tab PO once per week

Disp: #12 (Twelve)

Prescriber Signature Prescriber DEA

**Signature DEA NO.**

**Refill** Three **Times**

**Address** Prescriber Office Address

Students will have a blank prescription and should take a few minutes to try to do this on their own. This is one of the first times they have been asked to do this, so they will have some difficulty. Give them a few minutes and then discuss as a group. Some things to discuss:

- The importance of writing the full name of the patient (for security)
- The importance of a second identifier like the DOB
- The drug along with the strength of the tablet (if it’s a tablet) as well as how many tablets to take with each administration. For example, you might prescribe 10 mg tabs and have the patient take one in the morning and two at bedtime…)
- The total number of tablets (or other) to dispense
- Numbers for dispensation and refills are spelled out (to avoid tampering with amounts)
- “PO” means orally (per os), could write “by mouth” instead
- “QD” and “QOD” no longer ok – must write out “daily” or “every other day,” in this case, weekly

**PART 8**

**Ms. Schick is evaluated in the Emergency Room by the Orthopedic Surgery team. Because there is no evidence of neurovascular compromise, she is felt to be a good candidate for closed reduction.**

**A hematoma block is performed for analgesia (lidocaine is injected directly into the fracture hematoma) and then traction is applied (as shown).**

**Manual reduction is performed, and adequate alignment is achieved. A “sugar-tong” splint is applied (as shown) to avoid circumferential compression which would increase the risk of distal ischemia and carpal tunnel syndrome.**

**Ms. Schick is instructed to follow up in the Orthopedic Surgery clinic in two days for follow up X-rays and continued management. You explain to her that she should expect to be in the splint (or a smaller cast) for approximately six to eight weeks and that she will likely require physical therapy after the cast is removed in order to regain complete function.**


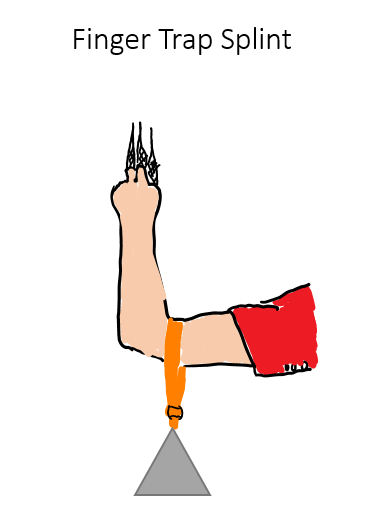
**In addition, Ms. Schick is instructed to follow up with her primary care physician with regards to further treatment for osteoporosis. She is unhappy about the events of the day, but very happy that you were the doctor on call in the Emergency Room!**


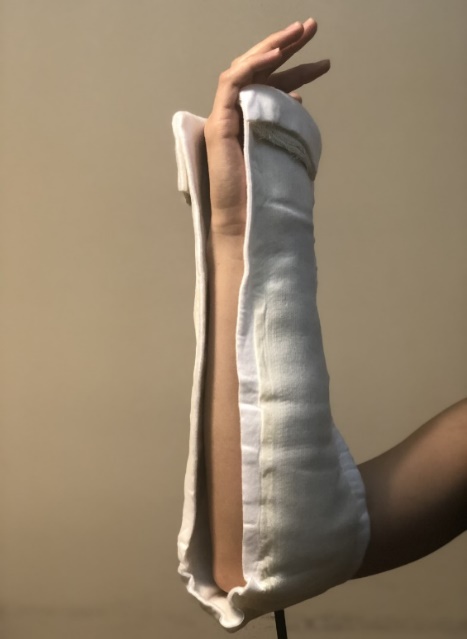


Sugar Tong Splint

Sugar tong forearm splints are indicated for distal radius and ulnar fractures. This flexible splint prevents motion of broken bones and immobilizes the elbow while allowing for expansion in the case of arm swelling. It is made from pre-fabricated material and maintains the wrist in a neutral position. *Image is author owned.*

Closed reduction and casting are treatments for distal forearm fractures. Finger trap fixtures are applied to three fingers and the limb is suspended for longitudinal traction. The elbow is bent at 90 degrees and upper arm is held perpendicular from floor. Gravity acting on weight supplies counter-traction to forearm. *Image is author owned.*

**EDUCATIONAL OBJECTIVES**

1. Perform or observe an oral case presentation and complete a musculoskeletal physical exam after obtaining hypothesis driven medical history.
2. Given a history of an acute injury to the wrist, use exam and x-ray appearance to diagnose a Colles type distal radial fracture.
3. Define a fragility fracture and recognize osteoporosis as the most common underlying cause.
4. When provided with a DXA result and T-Score, interpret the results of a DXA scan and understand the meaning of T-Score ranges.
5. Use the FRAX tool to predict the ten-year risk of osteoporotic fracture in a patient.
6. Name the available pharmacologic treatments for osteoporosis and describe the mechanism of action and major adverse effects of each.
