# Supplementary material for: Osteoporosis: A Small-Group Case-Based Learning Activity
Source: MedEdPORTAL. 2021 Aug 30;17:11176. doi: 10.15766/mep_2374-8265.11176 (PMC8403690; doi:10.15766/mep_2374-8265.11176)
Supplement: Supplementary file 1 — CBL Facilitator Guide.docxFace-to-Face Session Student Guide.docxRemote Learning Session Guide.pptxExam Question Descriptions.docxPostsession Survey.docx [file mep_2374-8265.11176-s001.zip › B. Face-to-Face Session Student Guide.docx]

**Student Guide**

**Ms. Schick**

**PART 1**

**Ms. Schick is a 69-year-old woman presenting to the Emergency Room with right wrist pain after a fall.**

**Q1. Is there any additional information that you want to ask of Ms. Schick? In addition to the group scribe, please take notes individually. After the history is obtained, one student will be asked to orally present the information that has been gathered.**

**PART 2**

**HPI**

Sophie Schick is a 69-year-old woman with a history of hypertension who presents with right wrist pain after a mechanical fall. Earlier today, she tripped over a parking stop in a grocery store parking lot, falling on her outstretched right arm. She landed on her right side, striking her hip, but did not hit her head. She has some pain at the hip, but has no trouble walking or weight bearing. She felt fine before the fall, denies dizziness or lightheadedness, and did not lose consciousness before or during the event. She felt immediate severe 10/10 pain in her right wrist which has now subsided to a 5/10. The pain is localized to the distal end of the right forearm, but radiates down to the fingers and up to the elbow. She is unable to move her wrist or fingers due to the pain, but denies paresthesia.

**PMH**

She has a history of Hypertension.

She has no known history of osteoporosis or osteopenia – she was told by her doctor several years ago that a bone test was “normal.”

**PSH**

She had a C-section for the birth of her son.

**MEDS**

She takes a medication for hypertension. She does not recall the full name, but it sounds like it is probably hydrochlorothiazide. She also takes a multivitamin sporadically but no supplemental calcium or Vitamin D.

**ALLERGIES**

No known drug allergies

**SH**

She is married and lives with her husband. Her son and his family live nearby. She worked as a 6^th^ grade math teacher but retired last year. She smokes about ½ a pack of cigarettes daily and has done so on and off since her 20s, but is interested in quitting. She drinks about 1 glass of wine per week with dinner and has no history of drug use. Her diet consists primarily of prepared foods and while she is active, she does not do any form of regular exercise.

**FH**

Her mother died in her 70s from a stroke that occurred during a prolonged and complicated recovery from a hip fracture. Her father also died in his 70s from pneumonia.

**Q2. Which parts of the focused physical exam would you perform? Why?**

**PART 3**

**Physical Examination**

Vitals:

Temp: 37.6 C HR: 97 bpm RR: 16 bpm BP: 142/84 SaO_2_ 98% on Room Air Weight: 53 kg Height: 5’3”

General: Pleasant, thin woman sitting up on the edge of the stretcher in mild-moderate pain.

HEENT: Atraumatic. Pupils equal, round, and reactive to light and accommodation. Sclerae clear, conjunctivae pink. Mucus membranes moist.

Chest: Clear to auscultation bilaterally.

CV: Normal S1S2, no murmurs/rubs/gallops.

Skin: Warm and dry. Scattered excoriations over the palms bilaterally and ecchymoses seen over the right hip.

Neuro: Alert and oriented, CN II-XII intact, normal strength and tone in all extremities except RUE, normal gait.

MSK: Thin with low-normal muscle bulk. Except for RUE, all joints have full range of motion without swelling, tenderness or erythema. There is no point tenderness to palpation along the length of the spine and no obvious kyphosis or loss of height. The RUE is normal above the wrist, including the elbow. Joints of the hand (MCPs, DIPs, and PIPs) are normal. There is no tenderness to palpation of the “anatomic snuffbox.” Just proximal to the wrist, the arm is warm, edematous, and grossly deformed as shown below:


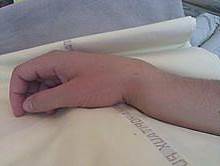


Image by Sylvian Letuffe, retrieved from: <https://commons.wikimedia.org/wiki/File:Poignet_Gauche_suite_a_fracture_type_Pouteau_Colles.jpg> on 12/11/2018. Image is in the public domain.

**Q3. How do you interpret the results of the physical exam?**

**Q4. What is the significance of the fact that there was no tenderness to palpation of the “anatomic snuffbox?” What are the borders of the “anatomic snuffbox?”**

**Q5. What type of injury did Ms. Schick most likely incur by falling on her extended wrist? Describe some of the clinical characteristics suggestive of the injury.**

**Q6. What if Ms. Schick had flexed her wrist prior to falling, which other type of injury might you expect her to have sustained?**

**Q7. Based on the history and physical exam, what other studies/interventions might you initiate in the ER today and why?**

**PART 4**

**Plain radiographs of the right wrist are performed (as seen). Radiographs of the right hip were also performed and showed no evidence of fracture. A CT of the head was performed and showed no evidence of trauma or intracranial hemorrhage.**


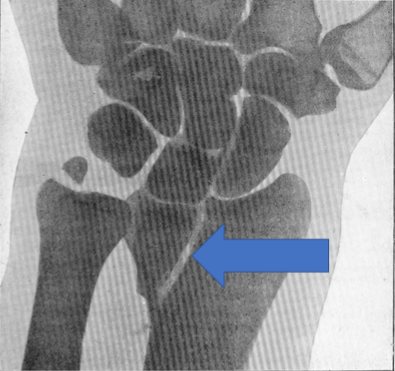


Image by Wikimedia Commons retrieved from: <https://commons.wikimedia.org/w/index.php?curid=36355446> on 11/03/2020. Creative Commons License associated: <https://creativecommons.org/licenses/by/4.0/deed.en>

**Q8. How do you interpret the x-ray? What type of fracture did Ms. Schick sustain? Use descriptive words to accurately describe the injury (i.e. open vs. closed, intra- vs. extra-articular, displaced vs. non-displaced, transverse vs. oblique vs. comminuted, etc.).**

**Q9. Based on the clinical presentation, are you concerned that Ms. Schick may have suffered a pathologic fracture? If so, which type is most likely? Define the term and explain your answer. In order to further investigate, which test(s) would you recommend that her primary care physician perform?**

**PART 5**

**Ms. Schick tells you that she is pretty sure her doctor did similar tests three years ago. Her husband brings the following results from home (shown below).**


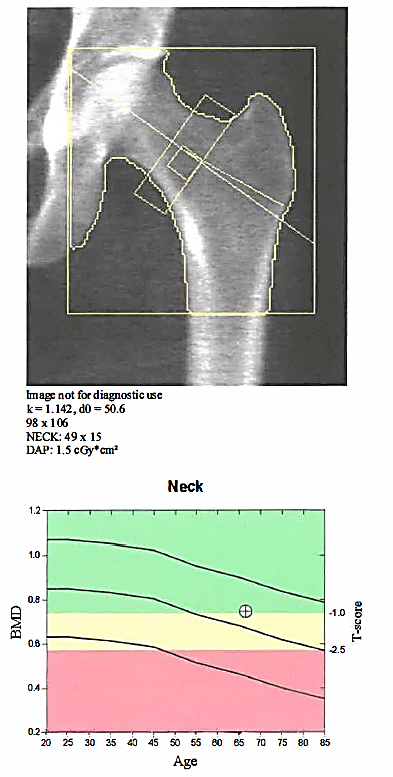
**
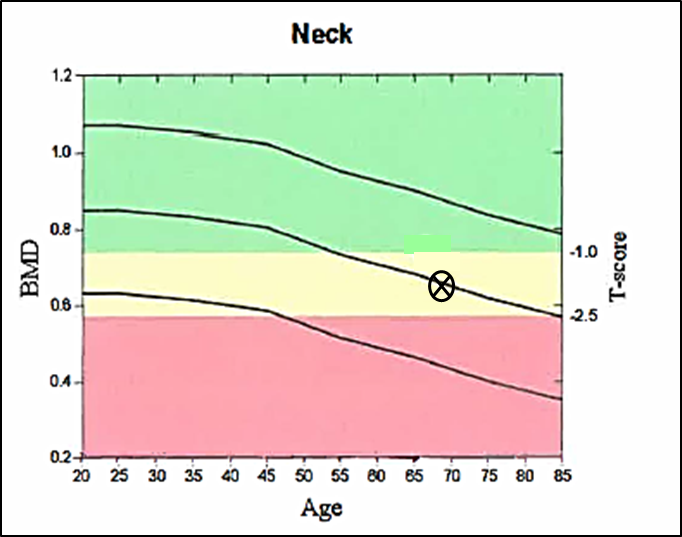
**

1. Author owned.

2. Author owned.

**Q10. What type of test is this? How do you interpret the results? What was Ms. Schick’s T-score? What does that mean?**

**Q11. Does Ms. Schick have osteoporosis? Did she have osteoporosis three years ago? Should she have received treatment three years ago? Why or why not?**

**PART 6**

**Q12. In retrospect, what risk factors did Ms. Schick have for an osteoporotic fracture? What are some other common risk factors?**

**Q13. In addition to the distal radius, Ms. Schick is at increased risk for fractures at which other sites?**

**Q14. How would you recommend that Ms. Schick be treated? If that includes pharmacologic interventions, be sure to describe the mechanism of action of the first line pharmacologic treatment and any important adverse effects and/or important patient instructions.**

**Q15. What other (second- or third-line) pharmacologic treatment options might you consider? Be sure to describe the mechanism of action of each.**

**Q16. In addition to pharmacologic interventions, what could Ms. Schick have done to mitigate her risk of sustaining an osteoporotic fracture? What factors will you tell her to consider now? Consider lifestyle choices as well as other environmental factors.**

**Q17. Please generate a problem list for Ms. Schick. Take a moment to do this individually, and then discuss together as a group.**

**PART 7**

**You discuss treatment options and your recommendation for alendronate treatment with Ms. Schick. She expresses concerns about potential adverse effects, stating “I saw a report on breakdown of the jaw that can occur from medications used to treat osteoporosis. Is this the same medication?” When you tell her that it is, she enquires about the risk of this complication. In order to answer her question, you find a recent systematic review that addresses the risk of osteonecrosis of the jaw with bisphosphonate treatment (data summarized below). The same data is presented below in a tabular format.**

| **Patient population** | **Incidence of Osteonecrosis of the Jaw** | | **ARD** | **NNH** |
| --- | --- | --- | --- | --- |
|  | **Receiving**  **Biphosphonates** | **Not Receiving Biphosphonates** |  |  |
| **Patients with *osteoporosis* receiving *oral* bisphosphonates** | 0.01% | 0.001% |  |  |
| **Patients with *cancer* receiving *intravenous* bisphosphonates** | 7.5% | 0.001% |  |  |

(For additional information, please refer to this article which was used to extract the data. Khosla S, Burr D, Cauley J,et al. [Bisphosphonate-associated osteonecrosis of the jaw: report of a task force of the American Society for Bone and Mineral Research.](https://www.ncbi.nlm.nih.gov/pubmed/17663640/)J Bone Miner Res. 2007 Oct;22(10):1479-91. doi: 10.1359/jbmr.0707onj. PubMed PMID: 17663640.)

**Q18. Calculate the absolute risk difference (ARD) and the number needed to harm (NNH) for the two patient populations included in this study. How would you interpret and present this data to Ms. Schick?**

**Q19. You decide to treat Ms. Schick with alendronate. Her DOB is 1/31/49. Using the prescription pad below, write a prescription for this medication. You may use electronic devices to help you decide upon a dose, frequency, and route of administration.**

**For** **Date**

**Address**


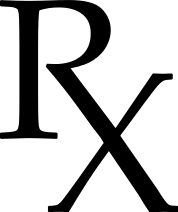


**Signature DEA NO.**

**Refill**   **Times**

**Address**

**PART 8**

**Ms. Schick is evaluated in the Emergency Room by the Orthopedic Surgery team. Because there is no evidence of neurovascular compromise, she is felt to be a good candidate for closed reduction.**

**A hematoma block is performed for analgesia (lidocaine is injected directly into the fracture hematoma) and then traction is applied (as shown).**

**Manual reduction is performed, and adequate alignment is achieved. A “sugar-tong” splint is applied (as shown) to avoid circumferential compression which would increase the risk of distal ischemia and carpal tunnel syndrome.**

**Ms. Schick is instructed to follow up in the Orthopedic Surgery clinic in two days for follow up X-rays and continued management. You explain to her that she should expect to be in the splint (or a smaller cast) for approximately six to eight weeks and that she will likely require physical therapy after the cast is removed in order to regain complete function.**


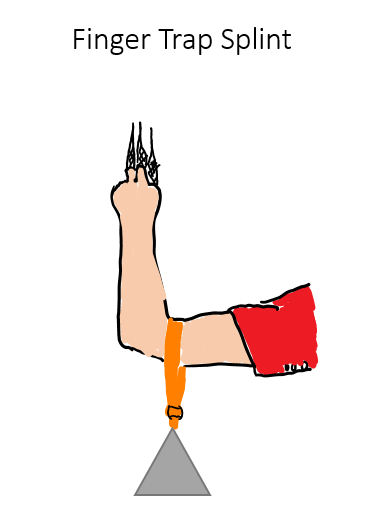
**In addition, Ms. Schick is instructed to follow up with her primary care physician with regards to further treatment for osteoporosis. She is unhappy about the events of the day, but very happy that you were the doctor on call in the Emergency Room!**


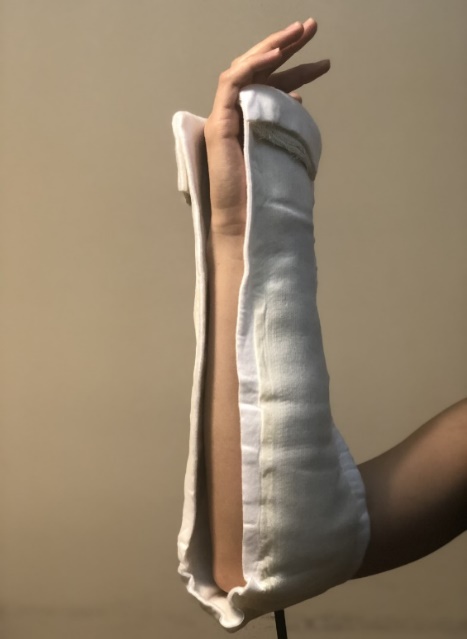


Sugar Tong Splint

Sugar tong splints are indicated for distal radius and ulnar fractures. This splint stabilizes forearm injuries by preventing wrist motion and forearm rotation. The splint, made from prefabricated splinting material, is long and U-shaped, and is used to maintain the alignment of broken bones. *Image is author owned.*

Closed reduction and casting provides treatment for distal forearm fractures. Finger trap fixtures are applied to three fingers, and the limb is suspended. Elbow is bent at 90 degrees and upper arm is straight out from the body. Gravity acting on weight supplies counter-traction to the forearm, which allows for muscle relaxation and restores arm length. *Image is author owned.*

**EDUCATIONAL OBJECTIVES**

1. Perform or observe an oral case presentation and complete a musculoskeletal physical exam after obtaining hypothesis driven medical history.
2. Given a history of an acute injury to the wrist, use exam and x-ray appearance to diagnose a Colles type distal radial fracture.
3. Define a fragility fracture and recognize osteoporosis as the most common underlying cause.
4. When provided with a DXA result and T-Score, interpret the results of a DXA scan and understand the meaning of T-Score ranges.
5. Use the FRAX tool to predict the ten-year risk of osteoporotic fracture in a patient.
6. Name the available pharmacologic treatments for osteoporosis and describe the mechanism of action and major adverse effects of each.
