# Supplementary material for: Osteoporosis: A Small-Group Case-Based Learning Activity
Source: MedEdPORTAL. 2021 Aug 30;17:11176. doi: 10.15766/mep_2374-8265.11176 (PMC8403690; doi:10.15766/mep_2374-8265.11176)
Supplement: Supplementary file 1 — CBL Facilitator Guide.docxFace-to-Face Session Student Guide.docxRemote Learning Session Guide.pptxExam Question Descriptions.docxPostsession Survey.docx [file mep_2374-8265.11176-s001.zip › D. Exam Question Descriptions.docx]

**Exam Question Descriptions**

1. Evaluation of osteoporosis treatment/prevention options
2. Recognize clinical presentation of osteoporosis
3. Mechanism of action/ drug interaction of osteoporosis medication
4. Identification of osteoporosis medication side effects
5. Identify risk factors of osteoporosis
6. Select the most suitable medications to treat osteoporosis
7. Interpret the results of the FRAX tool and the DXA scan
8. Review normal bone modeling/remodeling and factors regulating osteoclast activity
9. Identification of osteoporosis medication side effects
