# Supplementary material for: Osteoporosis: A Small-Group Case-Based Learning Activity
Source: MedEdPORTAL. 2021 Aug 30;17:11176. doi: 10.15766/mep_2374-8265.11176 (PMC8403690; doi:10.15766/mep_2374-8265.11176)
Supplement: Supplementary file 1 — CBL Facilitator Guide.docxFace-to-Face Session Student Guide.docxRemote Learning Session Guide.pptxExam Question Descriptions.docxPostsession Survey.docx [file mep_2374-8265.11176-s001.zip › E. Postsession Survey.docx]

**Post- Session Survey**

The basic science-clinical combination lecture on Osteoporosis followed by the small group case based discussion on Osteoporosis prepared me adequately to understand the topic.

- Strongly Agree
- Agree
- Neutral
- Disagree
- Strongly Disagree
