# Supplementary material for: Osteoporosis: A Small-Group Case-Based Learning Activity
Source: MedEdPORTAL. 2021 Aug 30;17:11176. doi: 10.15766/mep_2374-8265.11176 (PMC8403690; doi:10.15766/mep_2374-8265.11176)
Supplement: Supplementary file 1 — CBL Facilitator Guide.docxFace-to-Face Session Student Guide.docxRemote Learning Session Guide.pptxExam Question Descriptions.docxPostsession Survey.docx [file mep_2374-8265.11176-s001.zip › Educational_Summary_Report_11176.pdf]

MedEdPORTAL® The AAMC Journal of Teaching and Learning Resources

Original Publication Open Access

Osteoporosis: A Small-Group Case-Based Learning Activity

Bianca Nguyen, Gagani Athauda, MD, Sanaz B. Kashan, MD, Tracey Weiler, PhD, Rebecca L. Toonkel, MD*

*Corresponding author: rtoonkel@fiu.edu

Abstract

Introduction: Osteoporosis is the most common bone disease in the world. Approximately 50% of women and 20% of men over 50 will suffer an osteoporosis-related fracture. Future health care providers must be equipped to prevent, recognize, and treat osteoporosis-related fractures. **Methods:** To supplement instruction on osteoporosis, we designed a case-based session. Groups of 10-12 second-year medical students worked with a single facilitator in a roundtable discussion. The 120-minute session integrated foundational sciences (pathology, physiology, pharmacology) and clinical disciplines (clinical skills, radiology, geriatrics, evidence-based medicine). Knowledge gains were assessed by performance on nine session-relevant multiple-choice questions (MCQs) on the final exam. Student satisfaction was assessed by an anonymous postsession survey. **Results:** There were 121 students that participated, and their average performance on nine session-relevant final exam MCQs was 84%. After removal of a single outlier MCQ (15% correct), average performance on the remaining eight MCQs was 93%. A total of 107 students (88%) responded to the postsession survey. On a 5-point Likert scale, 101 of 107 students (94%) agreed or strongly agreed with the statement "The basic science-clinical combination lecture on osteoporosis followed by the small-group case discussion on osteoporosis prepared me adequately to understand the topic" ($M = 4.56$, $SD = 0.63$). **Discussion:** We developed a case-based learning activity for preclinical medical students to enhance the clinical scaffolding of basic science and medical knowledge around osteoporosis. Students performed well on session-relevant exam questions, demonstrating competency in the educational objectives. Student satisfaction was high, with most students feeling well prepared.

Keywords
Case-Based Learning, Problem-Based Learning, Osteoporosis, Osteopenia, Metabolic Bone Diseases, Colles' Fracture, Fracture Risk Assessment Tool, FRAX, Virtual Learning

Educational Objectives

By the end of this activity, learners will be able to:

1. Perform or observe an oral case presentation and complete a musculoskeletal physical exam after obtaining hypothesis-driven medical history.
2. When given a history of an acute injury to the wrist, use exam and X-ray appearance to diagnose a Colles-type distal radial fracture.
3. Define a fragility fracture and recognize osteoporosis as the most common underlying cause.
4. When provided with a bone density test (DXA) result and T-score, interpret the results of a DXA scan and understand the meaning of T-score ranges.
5. Use the Fracture Risk Assessment Tool to predict the 10-year risk of osteoporotic fracture in a patient.
6. Name the available pharmacologic treatments for osteoporosis and describe the mechanism of action and major adverse effects of each.

Citation:
Nguyen B, Athauda G, Kashan SB, Weiler T, Toonkel RL. Osteoporosis: a small-group case-based learning activity. *MedEdPORTAL*. 2021;17:11176.
https://doi.org/10.15766/mep_2374-8265.11176

Introduction

Osteoporosis, the most common bone disease in the world, leads to decreased bone strength, low bone mass, and increased risk of fractures.[1] An estimated 50% of women and 20% of men over the age of 50 will suffer an osteoporosis-related fracture, which is associated with disability, mortality, and significant financial cost to the patient and society.[1] As the population ages, the prevalence of osteoporosis and the number of associated fractures are expected to increase sharply.[1] Worldwide, the incidence of hip fractures is projected to increase dramatically in the coming decade, emphasizing the significance of prevention and treatment for osteoporosis-related injuries.[2] Future health care providers must be equipped to prevent, recognize, and treat osteoporosis-related fractures.

Copyright © 2021 Nguyen et al. This is an open-access publication distributed under the terms of the Creative Commons Attribution license.

Despite coverage by the USMLE Content Outline,[3] musculoskeletal (MSK) diseases like osteoporosis continue to be underrepresented in medical school curricula.[4] Because of this, the AAMC has voiced concerns that MSK medicine, particularly osteoporosis, may not be adequately emphasized in undergraduate medical education.[4]

A search of the literature revealed limited educational resources on osteoporosis appropriate for teaching preclinical medical students. At the time the search was conducted, there were four relevant *MedEdPORTAL* publications. One publication contains four virtual patient cases utilizing videos and images relating to the care of aging patients. One of these cases describes a geriatric patient with osteoporosis over a 15-year period, during which the patient develops additional health issues including hearing and vision loss, incontinence, hyperthyroidism, and cardiac disease.[5] *MedEdPORTAL* also has a publication on oral health describing endentulism and its associated morbidities, including the impact of oral health on diseases such as osteoporosis.[6] Another *MedEdPORTAL* publication describes short case studies on bone and muscle physiology.[7] While some of the cases involve osteopenia and osteoporosis, the activity primarily focuses on isolated basic sciences concepts rather than an integrated clinical approach including prevention, diagnosis, complications, and management. The fourth relevant *MedEdPORTAL* publication is a women's health tutorial consisting of 14 web-based modules with special emphasis on contraception, menopause, and preventive care.[8] The osteoporosis module consists of a PowerPoint presentation of content with two short cases for self-study.

A search of the PubMed and Google Scholar databases revealed a publication on the participation of health care providers and clinicians in a performance improvement continuing medical education initiative that includes eight 1-hour educational sessions to improve screening of patients at risk for osteoporosis.[9] Another study utilizes a questionnaire to assess the level of exposure of graduating family practice residents to fracture care.[10] A study carried out in Finland utilizes a questionnaire to assess the theoretical knowledge and Colles' fracture casting skills of graduating medical students.[11] Finally, there is a study that reports practice-based small-group learning interventions as an effective and acceptable method of providing continuing medical education on osteoporosis for primary care physicians.[12]

However, to our knowledge, there are no published cases designed to facilitate medical student integration of foundational science concepts, physical exam skills, evidence-based medicine, and clinical reasoning around osteoporosis. We sought to fill this unmet need at our institution through a small-group case-based learning (CBL) session designed to facilitate active learning and both horizontal and vertical integration of material across multiple courses and disciplines.

In contrast with previously published reports, our exercise focused directly on osteoporosis, from prevention through diagnosis and treatment.[5-12] This case was also designed to facilitate the progression of preclinical medical students toward attainment of several aspects of entrustability as outlined by the AAMC Core Entrustable Professional Activities (EPAs).[13] Through this exercise, students were prompted to obtain a focused history and perform the complete MSK exam (EPA 1: Gather a History and Perform a Physical Exam); recommend and interpret the results of laboratory studies, plain film radiography of a fracture, and a DXA (bone density test) scan (EPA 3: Recommend and Interpret Common Diagnostic and Screening Tests); write a safe and indicated prescription for a bisphosphonate (EPA 4: Enter and Discuss Orders and Prescriptions); orally present the history obtained from the patient (EPA 6: Provide an Oral Presentation of a Clinical Encounter); and use the Fracture Risk Assessment (FRAX) tool and evidence from the literature to decide on appropriate treatment for the individual patient (EPA 7: Form Clinical Questions and Retrieve Evidence to Advance Patient Care.)[14]

An additional advantage of our activity was its ability to be implemented remotely. As remote education on digital platforms continues to become more important, advances in computer technology and distance learning have enabled the remote integration of interactive teaching methods in medical education. Training a generation of future medical professionals who are digital natives entails incorporating novel learning tools and multimedia into the curriculum.[15] Through screen-sharing capabilities and remote conferencing platforms, small-group case-based sessions like ours can be easily implemented. Because of the difficulty in securing adequate small-group learning space in some institutions, the use of remote learning for these types of sessions is resource conserving and especially beneficial. In addition, emphasis on creative development of technology-enhanced learning tools will, through discussion with facilitators and peers, improve students' technological literacy and teamwork, which are necessary skills for clinical practice.[15]

Methods

We designed an interactive CBL session on osteoporosis intended for second-year medical students. At our institution, the

Copyright © 2021 Nguyen et al. This is an open-access publication distributed under the terms of the Creative Commons Attribution license.

session was implemented during the MSK systems course. Prior to the session, students had covered osteoporosis prevention, presentation, diagnosis, and treatment through an interactive didactic session co-led by an internal medicine specialist and a pharmacology expert. Students had also previously learned to obtain a focused history and to perform the MSK exam through their first-year clinical skills course.

Students were not expected to complete any preparatory assignments prior to the session; however, they were encouraged to be up to date in their studies in order to maximize their ability to participate effectively. Faculty facilitators were provided with the facilitator guide (Appendix A) a week in advance of the session and were expected to review the guide for flow and content (expected preparation time: approximately 30-60 minutes) but were not required to be content experts. There was no formal faculty development specific to this session, but all participating faculty were experienced CBL facilitators.

Traditional CBL sessions were conducted in small-group rooms (2015-2019) or via Zoom (2020), consisted of 10-12 students with a single faculty facilitator, and lasted 2 hours. We randomly preassigned learners to groups prior to the session. Case elements were progressively revealed through a hard-copy student guide (2015-2018; Appendix B), through a PowerPoint presentation projected in each room (2019; Appendix C), or via remote screen-sharing facilitated by Zoom (2020). Students worked together to answer the guided inquiry questions as the case elements were sequentially revealed. Discussions occurred organically, with answers provided by whichever student(s) chose to speak. Facilitators were discouraged from didactic teaching and instead prompted students with probing questions, managed time, maintained order, and ensured that each question was discussed thoroughly.

Effectiveness of the session was assessed by the inclusion of nine multiple-choice questions (MCQs) pertaining to osteoporosis management and treatment on the MSK medicine course final exam administered to the class of 2018. Participating teaching faculty in the MSK course designed the assessment items, which were edited by the members of the Exam Review Committee. Each question consisted of a single best answer format with four to five answer options. The committee reassesses and edits exam items annually based on student performance. Given the sensitive material in the assessment items, the questions have not been provided as part of this publication, but Appendix D contains descriptions of the areas the questions covered.

The average class performance and standard deviation were calculated for the nine osteoporosis-related MCQs. The item discrimination index, a form of a Spearman's correlation analysis that compared the performance of the overall exam to the performance of the individual items, was also calculated for each item. This analysis described how well the test question was able to differentiate between students who performed well on the exam overall and those who did not. Each exam item was associated with a learning objective covered by the CBL session. All data were deidentified by the Office of Testing and Assessment and provided to the investigators in aggregate.

Student satisfaction with the CBL session was evaluated through an anonymous postsession survey (Appendix E) administered to the class of 2018 immediately after the small-group CBL session. Students rated their level of agreement with the statement on a 5-point Likert scale (1 = *strongly disagree*, 2 = *disagree*, 3 = *neutral*, 4 = *agree*, 5 = *strongly agree*). The response rate and average statement agreement were calculated for the satisfaction survey.

This study received institutional review board exempt approval (#IRB-16-0086) from Florida International University.

Results

Average class performance on the nine osteoporosis-related final exam questions was 84% ($SD = 26\%$, $N = 121$; Table). With the exception of one question, greater than 90% of the class answered each of the questions correctly. Discrimination indices ranged from .01 to .23 ($M = .09$). One of the questions, which assessed risk factors for osteoporosis as defined by the FRAX tool, was answered correctly by only 15% of the class. When this outlier was removed, average class performance increased to 93% ($SD = 4\%$).

Response rate to the satisfaction survey was 88% (107 of 121 students). Average agreement with the statement "The basic science-clinical combination lecture on osteoporosis followed by the small-group case discussion on osteoporosis prepared me adequately to understand the topic" was 4.56 ($SD = 0.63$), with 94% (101 out of 107) agreeing or strongly agreeing (Figure).

Discussion

In alignment with recommendations from the Carnegie Foundation for the Advancement of Teaching, medical education continues to shift from teaching basic sciences in isolation toward the implementation of active learning pedagogies including CBL in order to encourage integration of curricular content and clinical application of knowledge.[16] The CBL session that we describe

Copyright © 2021 Nguyen et al. This is an open-access publication distributed under the terms of the Creative Commons Attribution license.

Table. Osteoporosis-Related Final Exam Performance (No. = 121)

Educational Objective(s) Assessed	Question Description	Item Difficulty	Discrimination Index
2, 4, 5, 6	Evaluation of osteoporosis treatment/prevention options.	.90	.12
1	Recognize clinical presentation of osteoporosis.	.90	.14
2, 5, 6	Mechanism of action/drug interaction of osteoporosis medication.	.93	.07
6	Identification of osteoporosis medication side effects.	.98	.02
3, 4	Identify risk factors of osteoporosis.	.15	.18
5, 6	Select the most suitable medications to treat osteoporosis.	.89	.01
2, 3, 4	Interpret the results of the FRAX tool and the DXA scan.	.88	.23
6	Review normal bone modeling/remodeling and factors regulating osteoclast activity.	.96	.05
6	Identification of osteoporosis medication side effects.	.98	.03
Overall average		.84	.09

Abbreviations: FRAX, Fracture Risk Assessment Tool; DXA, bone density test.

reflects these principles through the integration of foundational sciences and clinical disciplines.

As demonstrated by student performance on osteoporosis-related final exam questions, the activity was an effective teaching modality. Students performed well across the nine osteoporosis-related final exam questions. In response to poor student performance on a question regarding the FRAX tool, future iterations of the session included greater emphasis on the components of the FRAX tool and risk factors for osteoporosis.

Analysis revealed relatively low discrimination indices for each of the questions, suggesting that all students (both stronger and weaker students, based on their overall performance on the exam) were able to answer these questions correctly. A review of these questions with the course directors and subject matter experts revealed that they considered most of the questions to be fairly difficult. While this cannot be known without further study, it suggests that the combination of the didactic session and the CBL led to improved overall understanding and subsequent student outcomes on the assessment.

In addition, because no baseline assessment was performed either before or after the didactic session, it was unclear if students performed well on the osteoporosis-related questions

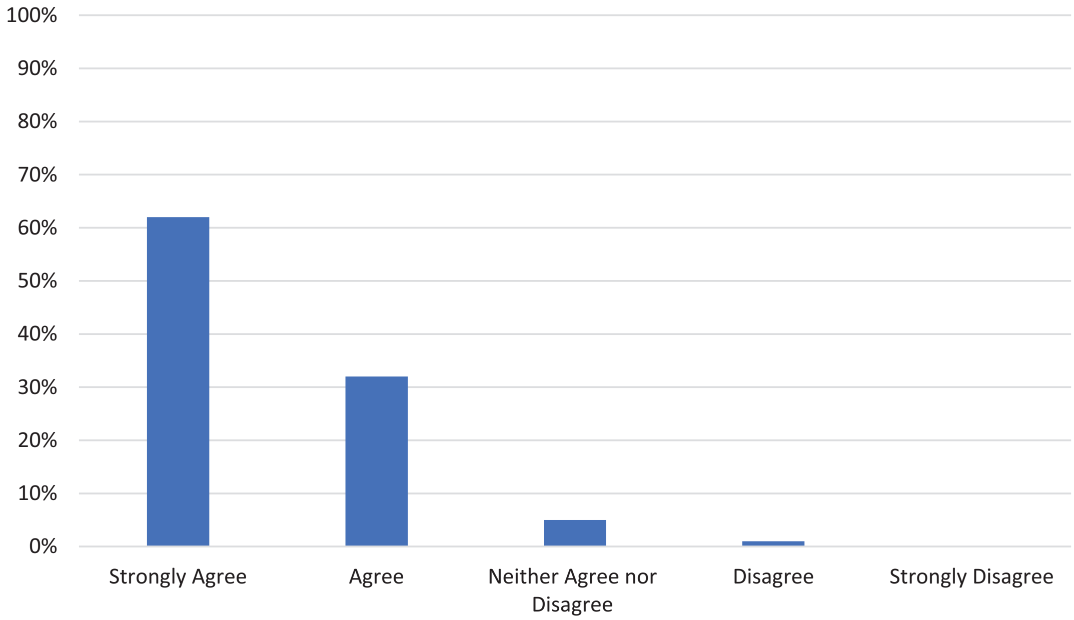

Figure. Student agreement with the statement “The basic science-clinical combination lecture on osteoporosis followed by the small-group case discussion on osteoporosis prepared me adequately to understand the topic” (*N* = 107).

Copyright © 2021 Nguyen et al. This is an open-access publication distributed under the terms of the Creative Commons Attribution license.

due to their intrinsic study skills (regardless of any teaching activities) or as a result of the didactic activity but not the CBL.

While student satisfaction does not reflect learning, it remains an important outcome with regard to student engagement and participation, which are crucial to the success of active learning pedagogies. For the case described, student satisfaction was high, suggesting that students felt that the session adequately prepared them to understand the topic. Future studies could include additional questions regarding satisfaction with the individual components of the teaching (e.g., the didactic session and the CBL) and could assess student perception of the value of the time spent in each of these activities.

In utilizing this activity for several years, we have identified a number of areas for potential improvement. For example, in this CBL, students were prompted to gather a history, perform a physical exam, order and interpret diagnostic tests, and incorporate epidemiologic concepts to optimize patient care. In order to fully engage in the activity, students were expected to review the material from their MSK, clinical skills, and evidence-based medicine courses prior to the session. While most students were able to perform these tasks, we did not perform a baseline readiness assessment. Educators who adapt this activity may improve student performance and participation by including a brief readiness assessment to ensure that students have reviewed all necessary material.

At our institution, prior knowledge of anatomy (skeletal system), physiology (normal bone functions), pharmacology (mechanism of action and adverse reactions of pharmacologic treatments), and clinical skills courses (history taking) was delivered in the first year. The evidence-based medicine concepts (absolute risk difference and number needed to harm) were taught during the second-year evidence-based medicine course. The topic of osteoporosis, including diagnosis, management, and treatment of the disease, was addressed during the second-year MSK and clinical skills courses.

As a result of the COVID-19 pandemic, the session transitioned to a virtual platform in 2020 using Zoom and Google Documents. To keep the students engaged with the virtual small-group CBL, they were instructed to keep cameras on during the active learning session and to use Google Documents to type answers to each question in an interactive format. Facilitators were asked to log into Zoom to ensure that their technology functioned properly, and they practiced sharing their slides before the session.

We developed a CBL session for second-year medical students aimed at integrating basic science concepts and clinical skills around osteoporosis. This session has undergone several iterations resulting in significant improvements that have been applied to other CBLs across the curriculum. In its most recent iteration, the case was adapted for live online learning with great success. As medical education moves increasingly toward active learning pedagogies, lessons learned from CBLs like this one will continue to gain importance. Especially for highly prevalent disorders like osteoporosis, CBLs provide an opportunity for students to scaffold medical knowledge with relevant clinical skills.

Appendices

A. CBL Facilitator Guide.docx
B. Face-to-Face Session Student Guide.docx
C. Remote Learning Session Guide.pptx
D. Exam Question Descriptions.docx
E. Postsession Survey.docx

All appendices are peer reviewed as integral parts of the Original Publication.

Bianca Nguyen: Medical Student, Florida International University Herbert Wertheim College of Medicine

Gagani Athauda, MD: Associate Professor, Department of Cellular Biology and Pharmacology, Florida International University Herbert Wertheim College of Medicine; ORCID: https://orcid.org/0000-0003-2920-4077

Sanaz B. Kashan, MD: Assistant Professor, Department of Humanities, Health, and Society, Florida International University Herbert Wertheim College of Medicine

Tracey Weiler, PhD: Associate Professor, Department of Human and Molecular Genetics, Florida International University Herbert Wertheim College of Medicine; ORCID: https://orcid.org/0000-0003-4662-4495

Rebecca L. Toonkel, MD: Associate Professor, Department of Translational Medicine, Florida International University Herbert Wertheim College of Medicine; ORCID: https://orcid.org/0000-0001-8339-2107

Acknowledgments
The authors gratefully acknowledge Jorge Perez, MSCP, MLIS, for help with image citations.

Disclosures
None to report.

Copyright © 2021 Nguyen et al. This is an open-access publication distributed under the terms of the Creative Commons Attribution license.

Funding/Support
None to report.

Ethical Approval
The Florida International University Institutional Review Board approved this project.

References

1. Coughlan T, Dockery F. Osteoporosis and fracture risk in older people. *Clin Med (Lond)*. 2014;14(2):187-191. https://doi.org/10.7861/clinmedicine.14-2-187
2. Pillai A, Eranki V, Shenoy R, Hadidi M. Age related incidence and early outcomes of hip fractures: a prospective cohort study of 1177 patients. *J Orthop Surg Res*. 2011;6:5. https://doi.org/10.1186/1749-799X-6-5
3. *USMLE Content Outline*. United States Medical Licensing Examination; 2021. Accessed May 28, 2021. https://www.usmle.org/pdfs/usmlecontentoutline.pdf
4. DiGiovanni BF, Sundem LT, Southgate RD, Lambert DR. Musculoskeletal medicine is underrepresented in the American medical school clinical curriculum. *Clin Orthop Relat Res*. 2016;474(4):901-907. https://doi.org/10.1007/s11999-015-4511-7
5. Denson K. Virtual patient case #4: Mrs. Violetta Mitsuko Tang—chief diagnosis: osteoporosis/incontinence. *MedEdPORTAL*. 2007;3:136. https://doi.org/10.15766/mep_2374-8265.136
6. Lee DJ, Felton DA. Oral health in medicine model curriculum: edentulism and complete denture care. *MedEdPORTAL*. 2012;8:9272. https://doi.org/10.15766/mep_2374-8265.9272
7. Hensley K. Mini case studies for bone and muscle physiology. *MedEdPORTAL,* 2010;6:8113. https://doi.org/10.15766/mep_2374-8265.8113
8. Davisson L, Nuss M, Bruno C, et al. Women's health tutorial. *MedEdPORTAL*. 2009;5:1060. https://doi.org/10.15766/mep_2374-8265.1060
9. Zisblatt L, Kues JR, Davis N, Willis CE. The long-term impact of a performance improvement continuing medical education intervention on osteoporosis screening. *J Contin Educ Health Prof*. 2013;33(4):206-214. https://doi.org/10.1002/chp.21200
10. Matheny JM, Brinker MR, Elliott MN, Blake R, Rowane MP. Confidence of graduating family practice residents in their management of musculoskeletal conditions. *Am J Orthop (Belle Mead NJ)*. 2000;29(12):945-952.
11. Helenius I, Sinisaari I, Hirvensalo E, Remes V. Surgical procedure skills of graduating medical students: effects of sex, working, and research experience. *J Surg Res*. 2002;102(2):178-184. https://doi.org/10.1006/jsre.2001.6310
12. Davis P, Andrews E, Donen N, et al. Case studies in osteoporosis: a problem based learning intervention for family physicians. *J Rheumatol*. 1999;26(11):2418-2422.
13. ten Cate O. Nuts and bolts of entrustable professional activities. *J Grad Med Educ*. 2013;5(1):157-158. https://doi.org/10.4300/JGME-D-12-00380.1
14. Obeso V, Brown D, Phillipi C, eds. *Core Entrustable Professional Activities for Entering Residency: Toolkits for the 13 Core EPAs*. Association of American Medical Colleges; 2017. https://www.aamc.org/media/20196/download
15. McCoy L, Lewis JH, Dalton D. Gamification and multimedia for medical education: a landscape review. *J Am Osteopath Assoc*. 2016;116(1):22-34. https://doi.org/10.7556/jaoa.2016.003
16. O'Brien BC, Irby DM. Enacting the Carnegie Foundation call for reform of medical school and residency. *Teach Learn Med*. 2013;25(suppl 1):S1-S8. https://doi.org/10.1080/10401334.2013.842915

Received: December 7, 2020
Accepted: May 25, 2021
Published: August 30, 2021

Copyright © 2021 Nguyen et al. This is an open-access publication distributed under the terms of the Creative Commons Attribution license.
